# Japanese Macaques’ (*Macaca fuscata*) sensitivity to human gaze and visual perspective in contexts of threat, cooperation, and competition

**DOI:** 10.1038/s41598-021-84250-5

**Published:** 2021-03-04

**Authors:** Alba Castellano-Navarro, Emilio Macanás-Martínez, Zhihong Xu, Federico Guillén-Salazar, Andrew J. J. MacIntosh, Federica Amici, Anna Albiach-Serrano

**Affiliations:** 1 Ethology and Animal Welfare Section, Universidad Cardenal Herrera-CEU, CEU Universities, Tirant lo Blanc 7, 46115 Alfara del Patriarca, Valencia Spain; 2 Primate Research Institute, Kyoto University, Kanrin 41-2, Inuyama, Aichi 484-8506 Japan; 3 Research Group Primate Behavioral Ecology, Department of Human Behavior, Ecology and Culture, Max-Planck Institute for Evolutionary Anthropology, Deutscher Platz 6, 04103 Leipzig, Germany; 4 Behavioral Ecology Research Group, Institute of Biology, Faculty of Life Science, University of Leipzig, Talstraße 33, 04103 Leipzig, Germany

**Keywords:** Animal behaviour, Psychology, Evolution, Anthropology, Evolutionary theory

## Abstract

Gaze sensitivity allows us to interpret the visual perspective of others, inferring their intentions and attentional states. In order to clarify the evolutionary history of this ability, we assessed the response of free-ranging Japanese macaques (*Macaca fuscata*) to human gaze in three contexts: threat (Experiment 1), cooperation (Experiment 2), and competition (Experiment 3). Subjects interpreted the direct gaze of an approaching human as a sign of threat, showing a greater flight initiation distance and more threats towards the human in this condition than when the human gazed in another direction. Subjects also adapted their behavior to the attentional cues of a human who gave them food, by for example moving into his visual field. However, the macaques did not seem to take the visual perspective of a human competing with them over food, as they failed to first retrieve the food that was not visible to the human (i.e., located behind an opaque barrier). Our results support the idea that Japanese macaques can respond to a human’s gaze flexibly depending on the context. Moreover, they highlight the importance of studying animal behavior across different species and contexts to better understand the selective pressures that might have led to its evolution.

## Introduction

Humans continually adjust their actions based on their interpretation of others’ intentions, desires, beliefs, etc. This capacity to attribute mental states to others is known as Theory of Mind (ToM). ToM is useful for explaining, predicting, and even manipulating others’ behavior, which is particularly advantageous in the case of highly social species. Since the first study of ToM with chimpanzees^1^, understanding the evolutionary origins of this cognitive capacity has been a key research directive. In particular, the field of comparative cognition has tried to answer this question by focusing not only on great apes, but also on other species more phylogenetically distant from ours^2,3^.

One of the most widely studied aspects of ToM in animals has been the ability to understand the visual perspective of others, which requires being sensitive to others’ gaze. Gaze is an important source of social information since, for example, it provides cues on the likelihood that others will respond to visual stimuli or on the direction of their next actions. Moreover, gaze is a window into others’ attentional states, intentions, visual perspective, knowledge and beliefs (including false beliefs). Therefore, being sensitive to gaze cues such as eye direction —although head and body orientation can also serve as useful approximations^4^— may confer direct fitness benefits in many different contexts. For example, the gaze of predators can signal danger to prey, favoring escape or avoidance behaviors^4–6^. Also, following others’ gaze may help to detect objects of interest, which is useful in both cooperative contexts (e.g., group hunting^7^) and competitive ones (e.g., competition over food^8^).

To date, many studies have assessed gaze sensitivity in primates and other vertebrates, and how the response to gaze varies depending on the context^9–11^. Evidence that animals understand the visual perspectives of others (i.e., what others can see from their own position), however, is much harder to obtain. In particular, it is difficult to design experimental paradigms that completely preclude the possibility that subjects respond by simply "reading" others’ behavioral cues (such as body orientation, head or eye direction), rather than responding to the representation of others’ visual perceptions. One experimental approach to the study of gaze sensitivity consists of a human approaching an animal in a threatening context while displaying different attentional states. In birds and reptiles, individuals flee faster and more often when the human displays a direct gaze toward the individual, as compared to an averted gaze toward a neutral point (e.g., ibis, *Bostrychia hagedash*^12^; iguanas, *Ctenosaura similis*^13^; crows, *Corvus brachyrhynchos*^14^; swallows, *Passer domesticus*^15^). As far as we know, only one study has so far assessed the response of a free-ranging primate (vervet monkey, *Chlorocebus pygerythrus*) to a human approaching^16^. In that study, however, the approaching human always faced the subject, so that nothing is yet known about primate abilities to use human gaze cues to adjust their flight behavior. Nevertheless, when tested with a similar paradigm designed to study the fearful temperament in laboratory primates (i.e., human intruder test), captive rhesus macaques (*Macaca mulatta*) responded with freezing behavior to an unfamiliar human standing in proximity but averting his/her gaze, whereas they displayed more aggressive bark vocalizations when the human’s gaze was directed to them^17^.

Gaze sensitivity has also been studied in cooperative contexts, mainly with two types of experimental paradigms. In object-choice tasks, the subject can choose from various containers, one of which contains food. A human experimenter indicates its location with his/her eyes or head orientation, or by pointing to the container with his/her arm, and then the subject can use these cues to find the food. Primates often perform rather poorly and inconsistently in these tasks (e.g., chimpanzees, *Pan troglodytes*^18–20^; bonobos, *Pan paniscus*^19^*;* orangutans, *Pongo pygmaeus*^19^; gorillas, *Gorilla gorilla*^21^; rhesus macaques^22^; snub-nosed monkeys, *Rhinopithecus roxellana*^23^; white-headed marmosets, *Saguinus oedipus*^24^). However, better performance is generally found in primates raised in close contact with humans (chimpanzees^25–27^, bonobos^27^, orangutans^28^), or after extensive training (capuchins, *Cebus apella*^29–31^).

The second experimental approach, known as food-requesting, conditional begging or donor-choice task, consists of testing whether subjects can use gaze cues to distinguish between the different attentional states of a human offering food, and accordingly adjust their response. Some studies have found that primates make more visual gestures toward the human when s/he is looking in their direction (e.g., squirrel monkey, *Saimiri sciureus*^32^; baboons, *Papio anubis*^33^), and more auditory signals when s/he is not (e.g., chimpanzees^34–36^; Japanese macaques, *Macaca fuscata*^37^). However, at least some primates may prefer to move and enter the human’s visual field when the human is looking in another direction, rather than using auditory signals (e.g., great apes^38^).

Studies using these cooperative paradigms suggest that primates can use human body and face orientation to guide their behavior, but they are not very sensitive to the direction of the human eye gaze or to the distinction between eyes-open and eyes-closed^22,39–41^. However, almost all these studies were carried out with captive primates trained to produce and interpret referential gestures, such as pointing, which do not belong to their natural repertoire^42,43^. In contrast, studies measuring spontaneous behaviors, such as time spent looking at the human (e.g., capuchins^44^), begging gestures (e.g., baboons^33^, capuchins^45^; chimpanzees^36^) or vocalizations (e.g., chimpanzees^36^), have shown that primates are able to distinguish between a human keeping his/her eyes open and closed. Therefore, testing free-ranging untrained individuals and observing their natural response appears especially informative. Furthermore, both cooperative paradigms mentioned above have been criticized because of their low ecological validity^46^, given that they require subjects to interact cooperatively with humans in order to obtain food, whereas, when food is at stake, most primates usually interact competitively with conspecifics^47–50^.

In line with this, Hare and colleagues^51^ designed an influential and more naturalistic paradigm, known as the food competition task. In this task, a subordinate and a dominant chimpanzee faced each other in two separate rooms. Between them was another room with two barriers, one opaque and one transparent, each with a piece of food placed on the side of the subordinate (so that the dominant could see the food behind the transparent barrier, but not the one behind the opaque barrier). The subordinate preferentially chose the food that the dominant could not see. This result was interpreted as evidence that chimpanzees understand the visual perspective of others (i.e., what the competitors can and cannot see from their position^51,52^).

Similar results were found in previous studies of macaques (Tonkean macaque, *Macaca tokeana*^53^; crab-eating macaque, *Macaca fascicularis*^54^), capuchin monkeys^55^, and marmosets (*Callithrix jacchus*^56^). However, it is also possible that subordinates were simply reacting to the dominants’ behavior (including, perhaps, the direction of their body, head or eyes), rather than representing their visual perspective^57,58^. Some studies have developed sophisticated controls to rule out this possibility^52^. Another option is to use a human as the competitor, whose behavior is easier to control. In a study of free-ranging rhesus macaques, Flombaum and Santos^59^ placed food in front of two human experimenters, one looking at the food and the other with his/her eyes closed (or covered), with the head turned or with the back to the food. The macaques preferred to grab the food located in front of the human who was not paying attention. Similar results have been found with various primate species in captivity, including chimpanzees^60^, gibbons (*Hoolock leuconedys* and *Hylobates Moloch*^61^), and lemurs (*Eulemur macaco, E. mongoz, Varecia variegata*, *E. fulvus*, *Propithecus coquereli*, and *Lemur catta*^62^).

In order to shed light into the evolutionary origins of ToM, we assessed gaze sensitivity in Japanese macaques, a primate species which is phylogenetically distant from ours and whose ToM-related cognitive abilities have not yet been extensively studied. In particular, we aimed to assess whether Japanese macaques 1) are sensitive to the human gaze and 2) can represent others’ visual perspectives. To explore whether specific selective pressures could have favored the evolution of these abilities, we tested the macaques in three different contexts: a context of threat in which an unfamiliar human approached them while either directly looking at them or looking at the ground (Experiment 1), a cooperative context in which a human gave them food showing different attentional states (Experiment 2), and a context in which they had to compete with a human by choosing the food that was visible or not visible to the human (Experiment 3). In the first two experiments we especially focused on subjects’ sensitivity to different visual gaze cues given by the experimenter. In the last experiment, we controlled for the possibility that macaques simply responded to visual gaze cues, and therefore tested for visual perception representation.

Based on previous studies that explored these contexts with other primate species, we predicted that Japanese macaques would be sensitive to the human gaze and other attentional cues in the threat context (Hypothesis 1) and in the cooperative context (Hypothesis 2) and would adjust their behavior according to the human visual perspective in the competitive context (Hypothesis 3). In particular, we expected that in the context of threat (Experiment 1) the macaques would flee earlier (i.e., at a greater distance from the human) and show more self-directed behaviors (SDB, a measure of anxiety in primates^63^), alarm calls, and threats towards the human when the human looked directly at them, as compared to when the human averted his gaze. In the context of cooperation (Experiment 2), given that the macaques could freely move, we predicted that they would move around the experimenter (entering his visual field) more frequently in the conditions in which the experimenter had his head turned or his back toward the macaques. Moreover, we expected more acoustic signs (e.g., vocalizations) and touching gestures (e.g., touches to the experimenter’s leg) when the experimenter had his eyes closed, his head turned or his back toward the macaques, and more visual gestures (e.g., begging gestures, threats) and SDB when the experimenter faced the macaques with his eyes open. In the context of competition (Experiment 3), we predicted that in the absence of human cues, the macaques would prefer to first reach for the food hidden from the human behind an opaque barrier, as compared to the visible food placed behind a transparent barrier.

We conducted this study with free-ranging macaques in their natural habitat, to ensure (1) that the results reflected species-typical abilities rather than an artifact of artificial rearing or housing conditions, and (2) a larger sample size, which allowed us to control for the effect of age, sex, and dominance rank on the individuals’ response to others’ gaze and visual perspective cues.

## Material and methods

### Ethics statement

All the protocols used in this study were approved by the Kushima Agency for Cultural Affairs and the ethic committee of Cooperative Research Program of Kyoto University’s Wildlife Research Center, following all relevant guidelines and regulations.

### Participants

In this study we tested Japanese macaques from a free-ranging population in Koshima (Miyazaki, Japan), an island of about 30 hectares almost completely covered by dense evergreen forest. The macaques of Koshima have been studied without interruption since 1948 and, since 1952, have been provided with wheat and/or sweet potatoes on a variable semi-daily basis^64^, currently twice per week. Therefore, all individuals of the main study group are habituated to the human presence, and their ages and sex are known.

Subjects were selected opportunistically when located away from the group in an open space free of obstacles. Forty-nine macaques participated in Experiment 1, 32 macaques in Experiment 2, and 31 macaques in Experiment 3 (Supplementary Table A.1). 

The dominance rank of the subjects was determined using the Elo method^65^. For a period of seven months, two investigators carried out ad libitum observations of the dyadic agonistic interactions in the group. We recorded all instances of unidirectional agonistic and submissive behaviors with a clear winner-loser outcome (Supplementary Table B.1). The position in the hierarchy was calculated with the “EloRating” package^66^ in R (version R 3.5.2.). Rank was determined for 51 individuals (32 females and 19 males; Supplementary Table A.1), but not for one of the male participants due to the few agonistic interactions in which this individual was involved.

### Procedure and data recording

#### Experiment 1: response to the gaze direction of a human in a threat context

**Figure 1:**
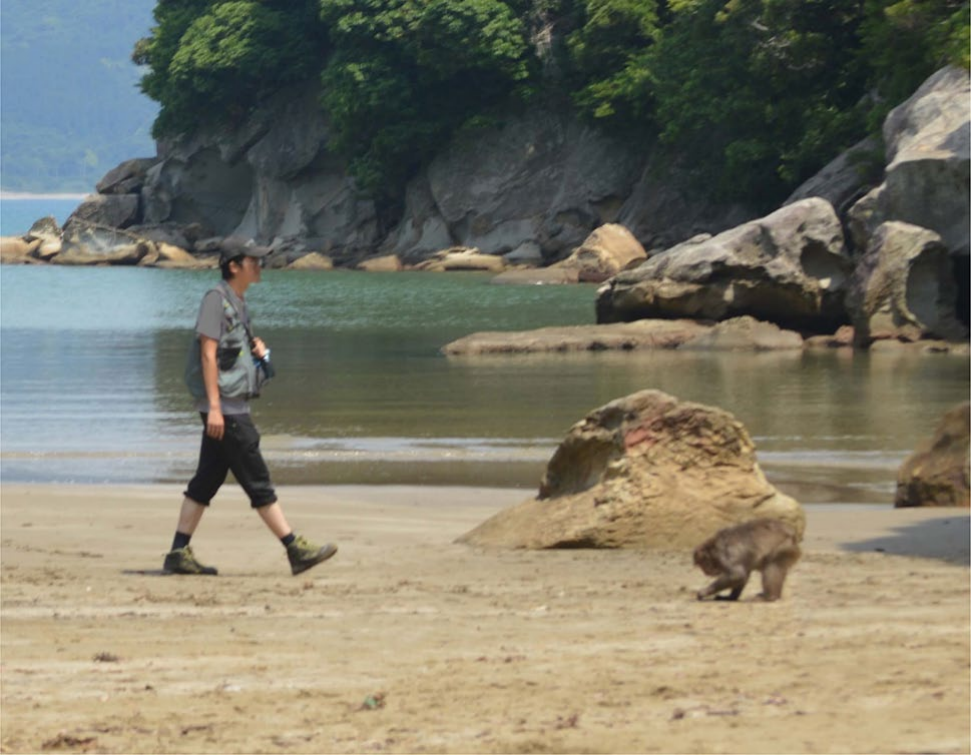
Position of experimenter 1 (E1) and subject (S) during an experimental trial in Experiment 1. E1 stands approximately 5 m in front of S, emits a soft vocalization so that S notices his presence and walks toward S at a constant speed (approximately 1 m/s). There were two conditions: Direct gaze towards S and Averted gaze towards the ground.

The first human experimenter (E1), a male who had not had previous contact with the macaques, placed himself approximately 5 m in front of the subject (S) and emitted a soft vocalization so that S would notice his presence. A trial started as soon as S looked at E1, at which point E1 walked toward S at a constant speed (approximately 1 m/s), maintaining a neutral expression (Fig. 1). There were two experimental conditions. In the first condition (Direct gaze condition), E1 oriented his eyes and face toward S. In the second condition (Averted gaze condition), E1 oriented his eyes and face to the ground. A trial ended when S moved away or, to avoid possible incidents, when E1 was 40 cm from S. A second experimenter (E2) stood at least 7 m from S, so as not to affect the target interaction, and recorded the trials with a camera. All subjects participated in 4 trials (two in each experimental condition), with an interval of at least 30 min between trials. The order of conditions was counter-balanced and pseudo-randomized across subjects.

We operationalized the “flight initiation distance” (FID) as the distance between the positions of E1 and S right before S moved away. We measured FID in situ with a measuring tape, recorded by E2. If the subjects did not flee when E1 reached a distance of 40 cm, it was assumed that they would withdraw when E1 took the next step, and so they were given an FID value of 0. From the videos recorded during the trials, E2 coded the number of threats to E1, alarm calls, and self-directed behaviors (i.e., SDB, scratching and self-grooming) per trial (Supplementary Table B.2).

#### Experiment 2: response to the attentional state of a human in a cooperative context

**Figure 2:**
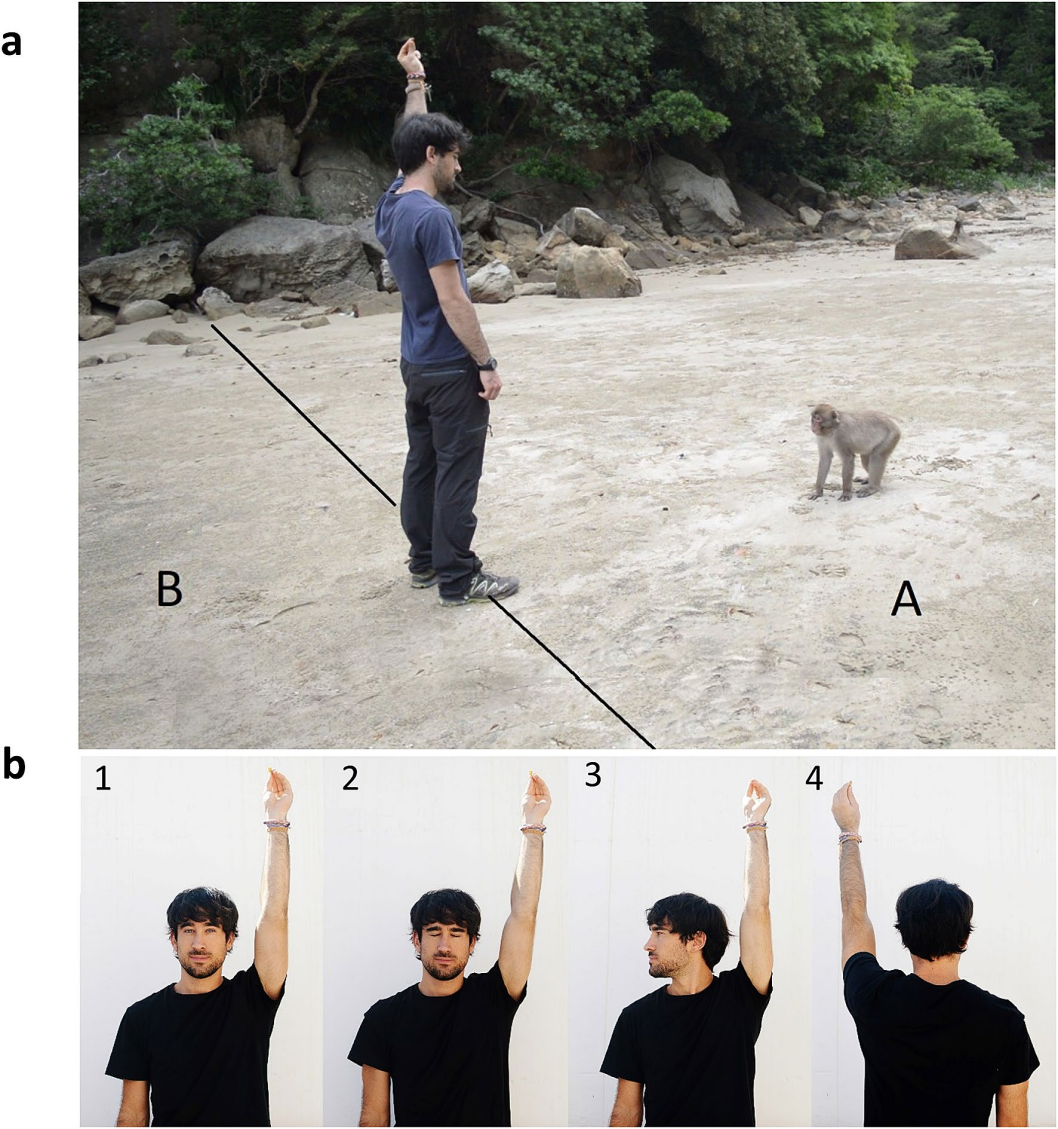
Set-up of Experiment 2. (**a**) Position of experimenter 1 (E1) and subject (S) at the beginning of each trial: E1 is approximately 2.5 m in front of S holding a peanut above its head. The imaginary line divides zones A and B where S can move during the test. (**b**) Experimental conditions: E1 holds a peanut over his head for 20 s with different states of attention: (1) Eyes open, (2) Eyes closed, (3) Head turned, (4) Back turned.

E1 (a familiar male experimenter, who had been working on the island for six months) was located facing S, approximately 2.5 m from S (Fig. 2a). Subjects were first administered one Familiarization trial, followed by 4 different Experimental trials. In the Familiarization trial, we aimed to show S that E1 had food and would give it to S. In this trial, E1 showed S a peanut (always with his left hand). As soon as S looked at the peanut, E1 raised the hand holding it over his head for 3 s and then threw it gently toward S, so that it fell approximately 2.5 m from E1.

In the Experimental trials, E1 showed a peanut to S, as above, and then raised the hand over his head for 20 s (to give the macaques enough time to respond). Depending on the condition, E1 could either (1) orient his body and face toward S, looking at S with his eyes open (Eyes open condition); (2) orient his body and face toward S, with his eyes closed (Eyes closed condition); (3) orient his body toward S, with his face turned 90° to the right (Head turned condition); or (4) orient his back toward S, looking forward (Back turned condition) (Fig. 2b). After the 20 s elapsed, E1 gently threw the peanut approximately 2.5 m from E1. E2, stood at least 7 m from S and recorded the trials with a video camera. All subjects received one trial per condition, with the order of conditions being counter-balanced and pseudo-randomized across subjects. An attempt was made to administer each S all trials in the same session. When this was not possible, the remaining trials were administered in a following session, always starting with a Familiarization trial. A total of 21 trials were not completed and had to be repeated due to the interference of other individuals (*n* = 19) or experimenter error (*n* = 2).

For each S and 20-s Experimental trial, E2 coded from the videos: the number of vocalizations, instances of S moving within E1′s visual field (i.e., moving to the left in the Head turned condition, and moving behind E1 in the Back turned condition), threats to E1, SDB (i.e., self-scratching), and touching gestures (e.g., touching E1′s leg; see Supplementary Table B.3).

#### Experiment 3: response to the visual perspective of a human in a competitive context

**Figure 3:**
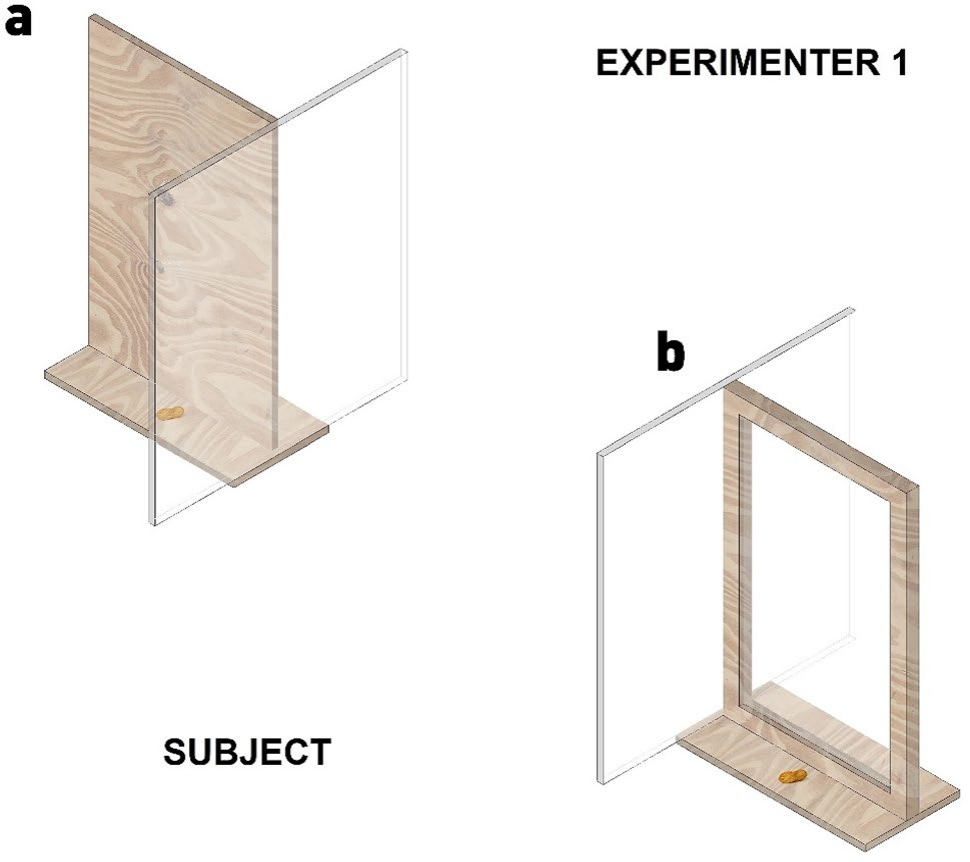
Apparatus used in Experiment 3: (**a**) opaque barrier, (**b**) transparent barrier.

In this experiment, we used an apparatus composed of two aligned rectangular barriers (29 × 45 cm). Each barrier was screwed to one horizontal wooden support (30 × 15 cm), whereon peanuts were placed. One of the barriers was opaque (made of wood), and the other was transparent (made of plexiglas), both surrounded by a wooden frame. A translucid plexiglas structure (40 × 45 cm), perpendicular to the lateral interior part of each barrier, prevented individuals from accessing the peanut from that side (Fig. 3).

The experiment consisted of two sessions, differing in the distance between the barriers (0.5 and 2 m, respectively), as a larger distance may decrease the chances that S obtains both food rewards and thus should increase its motivation to choose strategically^52^. Both sessions started with a Familiarization phase, immediately followed by an Experimental trial and a Control trial.

In the Familiarization phase, E1 (a familiar male experimenter), faced S at approximately 4 m distance. E2 threw a peanut between E1 and S. Immediately, E1 tried to retrieve the peanut before S, showing a threatening attitude (i.e., strong steps and rapid and abrupt movements towards food). We administered 3 trials in the first session, and 5 in the second one. The aim of this phase was to show E1′s interest in food and his competitive attitude towards S.

**Figure 4:**
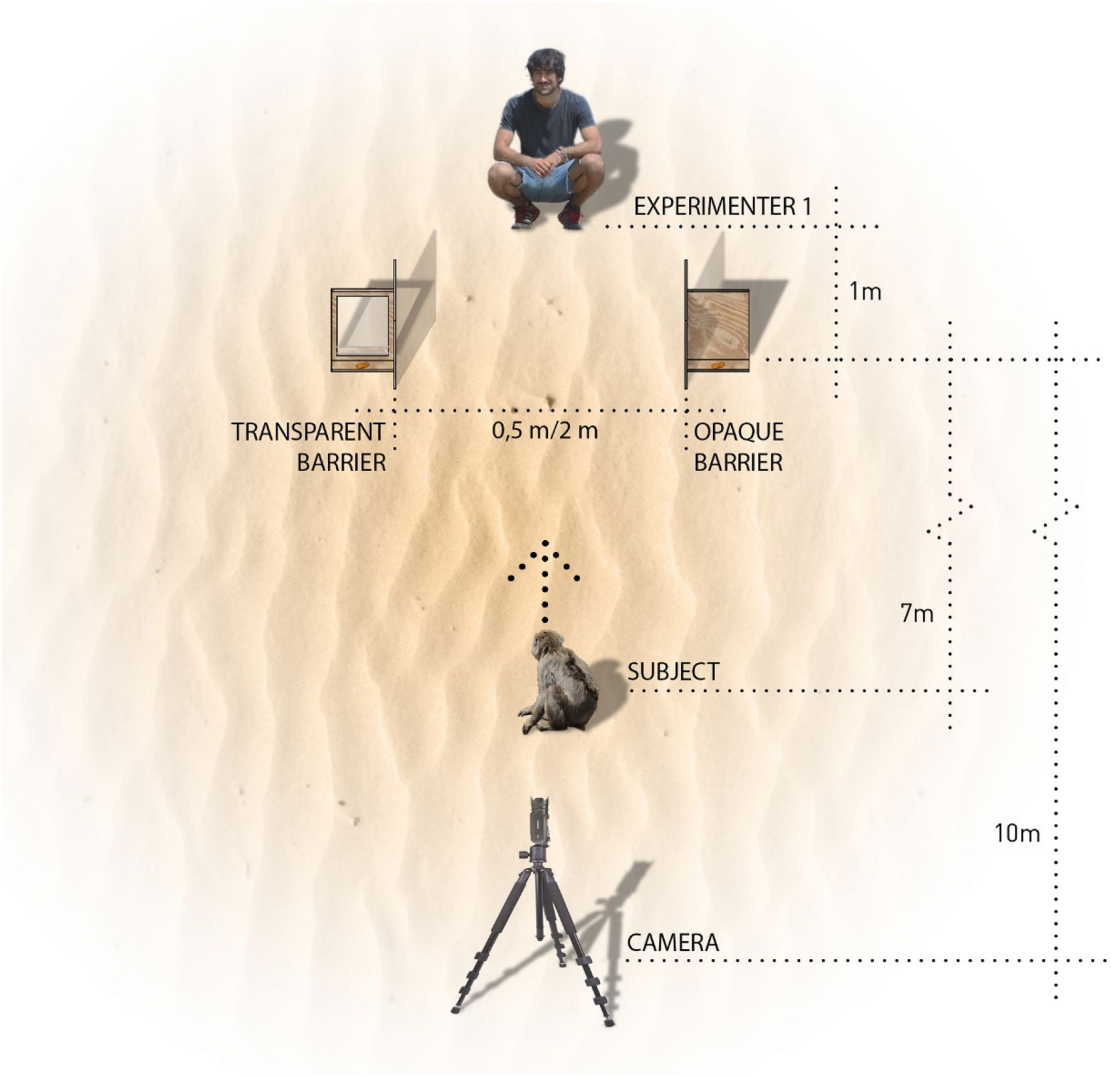
Scheme of the procedure used in the Experimental condition of Experiment 3. The opaque and transparent barriers, with a peanut in front of each one, are located between the experimenter 1 and the subject. The distance between both barriers is 0.5 m in the Session 1 and 2 m in Session 2. A camera on a tripod records the trials.

In the Experimental condition, E2 placed the barriers between E1 and S (Fig. 4). E1 was 1 m from the barriers, squatting, with a neutral facial expression and his eyes fixed on a point on the ground between the barriers. E2 placed a video camera on a tripod, 10 m from the barriers, behind S, and activated the recording. Then, E2 stood between the two barriers, looking at S, and threw a peanut in a straight line, about 7 m from the apparatus, so that S would stand there and eat the peanut. In the meantime, E2 placed a new peanut on each of the platforms (on S’s side), making sure that S saw it. After that, E2 quickly withdrew, not to interfere with the task. From the moment the two peanuts were placed on the platforms, experimenters waited 1 min for S to approach and take at least one of the peanuts. E1 did not move during this time, so S could take one peanut first, and then the other. In the Control condition, the procedure was identical, except that E1 was not present. This condition allowed assessing whether subjects preferred either of the two barriers, regardless of the presence of the human competitor.

Both the order of trials (i.e., Experimental and Control conditions) and the position of the barriers (opaque barrier to the S’s right or left) were counter-balanced and pseudo-randomized across subjects. A total of 41 trials could not be completed and had to be repeated due to the interference of other individuals (*n* = 27), the approach of S before the end of the presentation (*n* = 5), experimenter error (*n* = 4), and S’s refusal to participate in the task (*n* = 5). From the videos, E2 coded the first choice made by S in each trial (i.e., whether S took the peanut located behind the opaque barrier or behind the transparent one).

### Data analysis

From the videos of the three experiments, another researcher coded a 20% of the trials. Interrater reliability was good (minimum Cohen’s Kappa coefficient Experiment 1 = 0.787, *n* = 39; Experiment 2 = 0.833, *n* = 24; Experiment 3 = 1, *n* = 18).

Statistical analyses were run with a Bayesian approach, using the “rethinking” package^67^ in R (version 3.2.3). Table 1 summarizes the list of the models used in this study. In Experiment 1, we used a normal distribution to model FID as a continuous dependent variable (set M1) and a Poisson distribution to model the number of threats to E1 (set M2), alarm calls (set M3), and SDB (set M4). In Experiment 2, our dependent variables were the number of each of the recorded behaviors per each 20-s trial (i.e., vocalizations: set M5; moving to the left: set M6; moving behind E1: set M7; threats to E1: set M8; and SDB: set M9), with a Poisson distribution. As no touching gestures were performed by the macaques, we did not include models with this as a dependent variable. In Experiment 3, we included S’s first choice (opaque or transparent barrier) as the dependent variable (set M10), using a binomial distribution. In all the models, we added a varying intercept by S identity to correct for repeated observations, and S’s age, sex, and rank as fixed effects to control for interindividual differences. Due to convergence issues, we did not include the 2-way interactions between condition and individual characteristics in the models (so that we were not able to assess how individual characteristics like sex, age or rank modulate individuals’ responses in the different conditions), and neither did we include the trial number (although, given the low number of trials per condition ‒1 or 2‒, we consider unlikely that a learning effect occurred across trials).

**Table 1:** List of the models used in this study, including the dependent variable, the number of the model as used in the text, the fixed effects, the Widely Applicable Information Criteria (WAIC), and the Akaike weight of each model. The R^2^ shown corresponds to the best model in each set.

Set^a^: Dependent variable	Model^b^	Fixed effects included	WAIC	Weight	R^2^
**Exp. 1**					
1: Flight initiation distance (FID)	**M1.1**	**Condition**	**2351.9**	**0.88**	**0.63**
	M1.0	–	2356.0	0.12	
2: Num. threats to E1	**M2.1**	**Condition**	**101.5**	**0.65**	**0.23**
	M2.0	–	102.7	0.35	
3: Num. alarm calls	**M3.0**	**–**	**84.5**	**0.74**	**0.13**
	M3.1	Condition	86.6	0.26	
4: Num. self-directed behaviors (SDB)	**M4.0**	**–**	**168.6**	**0.66**	**0.02**
	M4.1	Condition	169.9	0.34	
**Exp. 2**					
5: Num. vocalizations	**M5.0**	**–**	**82.6**	**0.97**	**0.73**
	M5.1	Condition	89.0	0.03	
6: Num. moving to the left	**M6.1**	**Condition**	**123.7**	**1.00**	**0.21**
	M6.0	–	134.8	0.00	
7: Num. moving behind E1	**M7.1**	**Condition**	**73.4**	**0.52**	**0.24**
	M7.0	–	87.7	0.00	
8: Num. threats to E1	**M8.1**	**Condition**	**73.4**	**1.00**	**0.65**
	M8.0	–	111.0	0.00	
9: Num. self-directed behaviors	**M9.1**	**Condition**	**115.5**	**0.96**	**0.29**
	M9.0	–	122.0	0.04	
**Exp. 3**					
10: Choice of the opaque barrier	**M10.0**	**–**	**120.3**	**0.63**	**0.04**
	M10.1	Distance, condition	122.4	0.22	
	M10.2	Distance*condition	123.2	0.14	

Exp. = experiment, Num. = number, E1 = experimenter 1

^a^Each set of models has the same dependent variable.

^b^The models in each set are ordered from the lowest to the highest WAIC, and the best model is presented in bold and accompanied with its R^2^ coefficient. In all models, we also included a varying intercept by subject identity, as well as the fixed effect of subject’s age, sex, and rank as control.

We compared the null controls-only model (models M1.0 to M10.0) to a model obtained by adding the condition (i.e., Experiment 1: Direct gaze and Averted gaze; Experiment 2: Eyes open, Eyes closed, Head turned, and Back turned; Experiment 3: Experimental and Control) as a fixed effect (models M1.1 to M10.1) (see Table 1). In Experiment 3, we also included the fixed effect of distance between barriers (0.5 m and 2 m) in model M.10.1, and the 2-way interaction between condition and distance between barriers (model M10.2) (see Table 1).

To rule out collinearity, we determined the variance inflation factors (VIFs^68^), which were minimal (maximum VIF across all models = 1.91). In all models, we used weakly informative priors (normal [0,10] for predictors and Cauchy for random effects). We estimated parameters with RStan^69^, running 3 Hamiltonian Monte Carlo chains in parallel to draw more independent samples from our models and increase accurate inference^67^. Each chain included 100,000 samples, half of which were warm-up samples (i.e., samples that are not part of the posterior distribution, but are initially required to improve sampling efficiency^67^). Convergence was suggested by a generally high effective number of samples (mean n_eff across all models = 39,828, range 962–235,938) and Rhat estimates (which test if chains have mixed well by comparing the between- and within-chain estimates for the model parameters) of 1.00^67^. We then selected models based on the lowest Widely Applicable Information Criteria (i.e., WAIC, which estimates the average deviance on a new sample deviance and is thus a measure of the model accuracy^67^), and high Akaike weights (which are transformed information criterion values analogous to posterior probabilities of models and estimate the relative probability that different models will best predict future data^67^) (see Table 1). We finally used R^2^ to measure the proportion of variance explained by the model and thus assess model performance for the best model (see Table 1).

## Results

### Experiment 1

**Figure 5:**
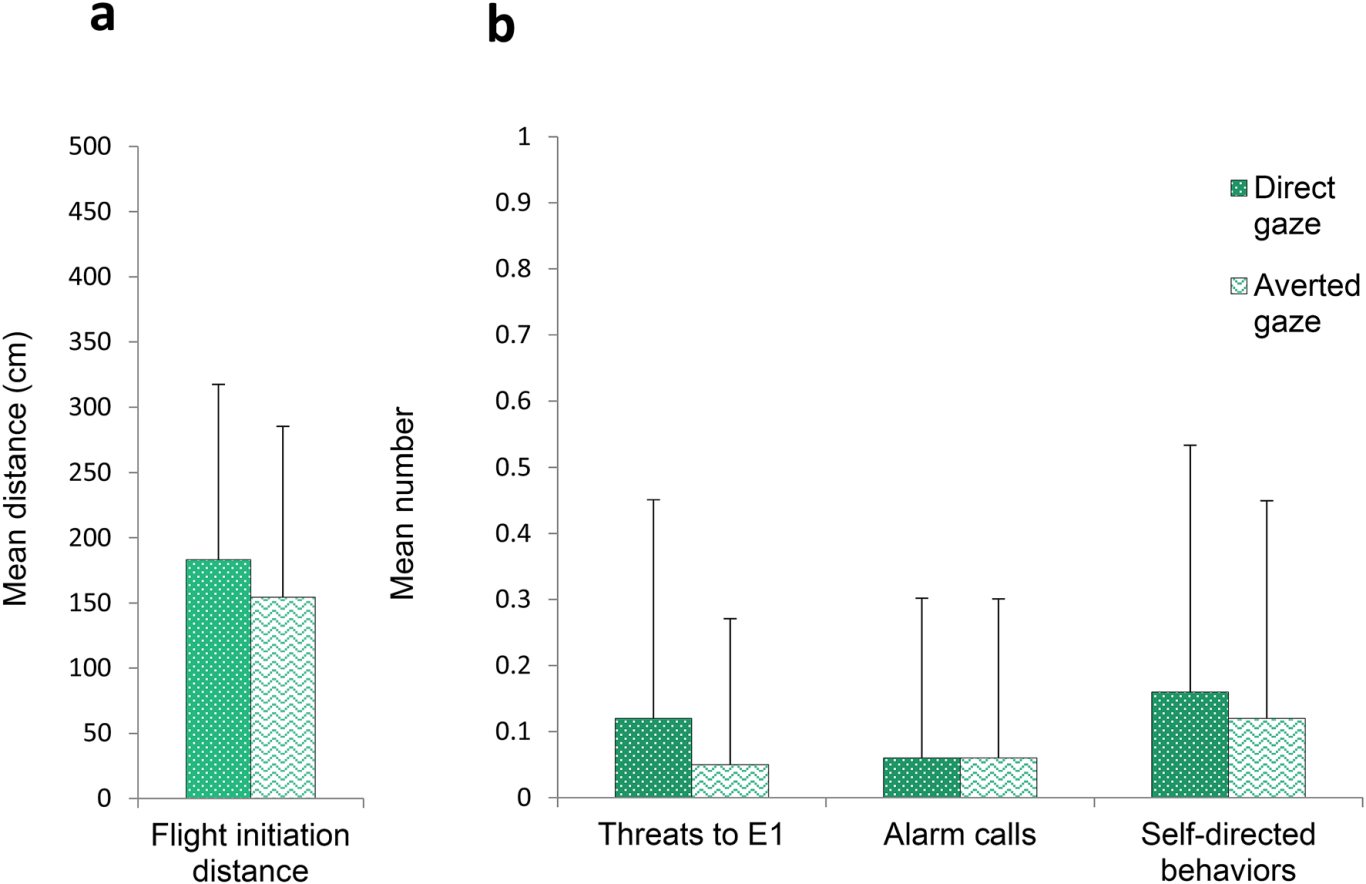
(**a**) Mean flight initiation distance (± *SD*) and (**b**) Mean number (± *SD*) of threats to experimenter 1 (E1), alarm calls and self-directed behaviors exhibited by the subjects in the Direct gaze and Averted gaze conditions of Experiment 1.

Figure 5 shows (a) the mean flight initiation distance (FID) ± *SD* and (b) the mean number of threats to experimenter 1 (E1), alarm calls, and self-directed behaviors (SDB) performed by the macaques in the Direct gaze condition and the Averted gaze condition. The subjects’ FID differed between conditions, depending on how E1 approached them (model M1.1 in Table 1, Fig. 5). In particular, FID was larger (i.e., subjects moved away earlier) when E1 approached them with a direct gaze as compared to an averted gaze (β = 15.74, 5.5–94.5% Prediction Interval [PI] = 3.09 to 28.33). The condition also predicted the subjects’ threatening behavior (model M2.1 in Table 1, Fig. 5), with subjects producing more threats towards E1 when E1 looked at them with a direct gaze as compared to an averted gaze (β = 0.93, 5.5–94.5% PI = 0.08 to 1.83). Finally, there were no differences in the number of alarm calls and SDB exhibited by the subjects in both conditions (models M3.0 and M4.0 in Table 1, Fig. 5).

### Experiment 2

**Figure 6:**
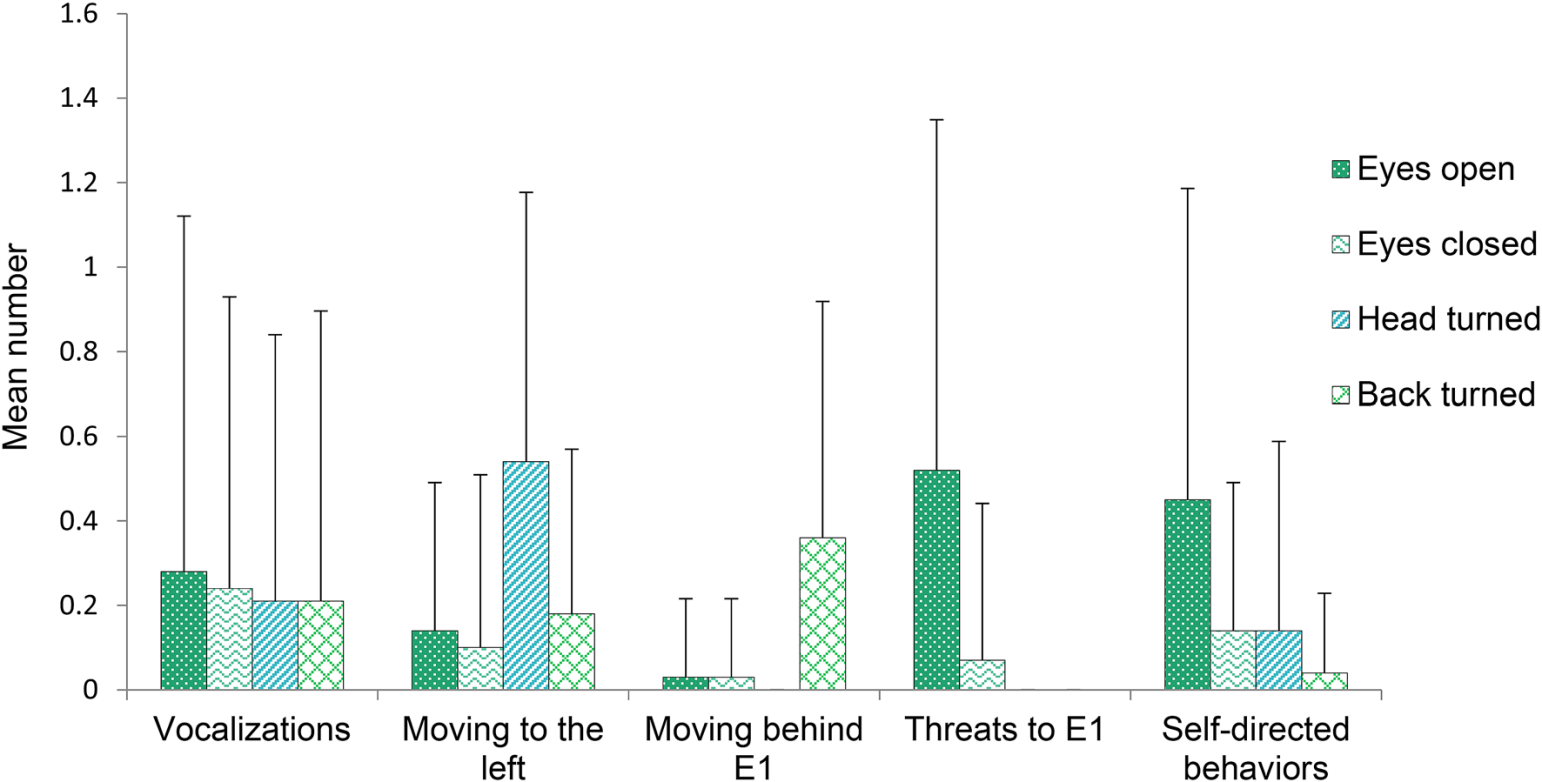
Mean number (± *SD*) of the behaviors exhibited by the subjects in the different conditions of Experiment 2.

Figure 6 shows the mean number of behaviors recorded in the 4 conditions (Eyes open, Eyes closed, Head turned and Back turned). Firstly, E1’s eyes, face, and body state had no effect on the probability of subjects’ vocalizations (model M5.0 in Table 1, Fig. 6). However, the probability of the other studied behaviors varied across conditions. Subjects moved to the left more frequently when E1 had his face turned in that direction (i.e., entering E1’s visual field) than in all the other conditions (Eyes open: β =  − 1.45, 5.5–94.5% PI =  − 2.45 to − 0.56; Eyes closed: β =  − 3.19, 5.5–94.5% PI =  − 5.40 to − 1.54; Back turned: β =  − 1.16, 5.5–94.5% PI =  − 2.05 to − 0.34; model M6.1 in Table 1, Fig. 6). In addition, subjects entered E1′s visual field by moving behind him more frequently when E1 had his back to the subject than in the other conditions (Eyes open: β =  − 2.80, 5.5–94.5% PI =  − 5.08 to − 1.09; Eyes closed: β =  − 2.72, 5.5–94.5% PI =  − 4.98 to − 1.02; Head turned: β =  − 9.75, 5.5–94.5% PI =  − 20.30 to − 2.89; model M7.1 in Table 1, Fig. 6). Moreover, threats towards E1 were more frequent when E1 was looking at subjects with his eyes open than in all the other conditions (Eyes closed: β =  − 2.23, 5.5–94.5% PI =  − 3.67 to − 1.03; Head turned: β =  − 10.02, 5.5–94.5% PI =  − 20.31 to − 3.32; Back turned: β =  − 10.05, 5.5–94.5% PI =  − 20.49 to − 3.29; model M8.1 in Table 1, Fig. 6). Lastly, subjects performed SDB more frequently when E1 looked directly at them than in all the other conditions (Eyes closed: β =  − 1.23, 5.5–94.5% PI =  − 2.24 to − 0.33; Head turned: β =  − 1.25, 5.5–94.5% PI =  − 2.27 to − 0.34; Back turned: β =  − 2.97, 5.5–94.5% PI =  − 5.21 to − 1.29; model M9.1 in Table 1, Fig. 6).

### Experiment 3

**Figure 7:**
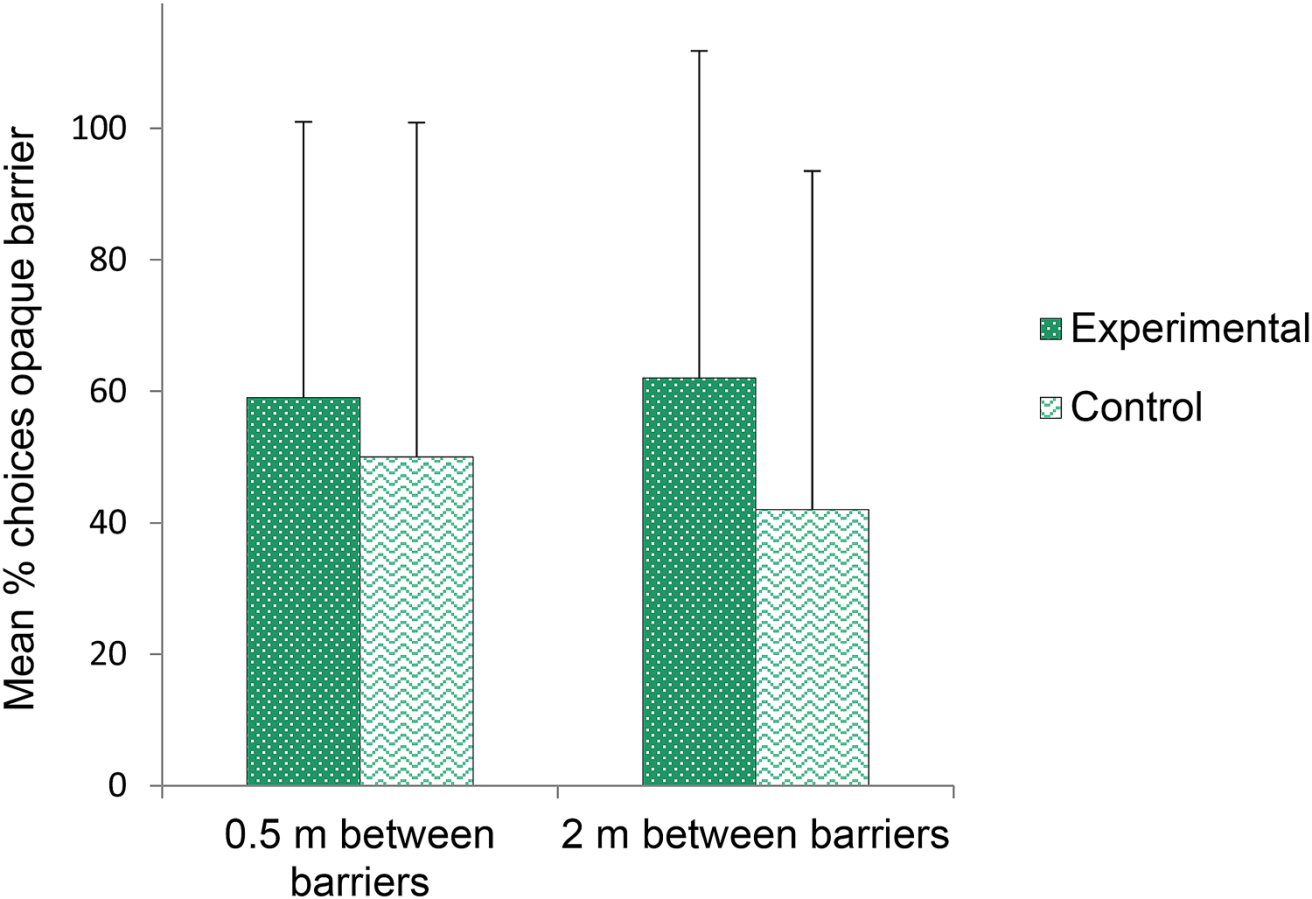
For each condition (Experimental and Control) and each distance between barriers (0.5 m and 2 m), mean percentage of trials (± *SD*) in which the subject first took the reward in front of the opaque barrier in Experiment 3.

Figure 7 shows the mean percentage of trials in which S first took the reward behind the opaque barrier, depending on the condition (Experimental and Control, i.e., presence and absence of E1) and the distance between the barriers (0.5 m and 2 m). In this experiment, condition, distance between barriers, and their 2-way interaction had no effect on subjects’ preference for one type of barrier (model M10.0 in Table 1, Fig. 7).

## Discussion

In this study, we assessed sensitivity to the human gaze and visual perspective in a group of free-ranging Japanese macaques. We carried out three experiments, each focusing on a different context: threat (Experiment 1), cooperation (Experiment 2), and competition (Experiment 3). Macaques were found to be sensitive to the attentional cues of a human in the two first experiments (Hypothesis 1 and 2 supported), but we found no evidence that they represented the visual perspective of the human in the last experiment (Hypothesis 3 not supported).

In the first two experiments, the subject (S) responded to the experimenter’s (E1) gaze cues differently depending on the situation. Macaques flight initiation distance (FID) and the number of threats towards E1 were larger when E1 approached while directly looking at them (Experiment 1), and macaques reliably moved to enter E1’s visual field when he had his head or body turned in another direction, increasing the probability of receiving the food he was offering (Experiment 2). Importantly, subjects reacted differently to the same cue (i.e., E1’s frontal face) in the two experiments, avoiding it in Experiment 1, while moving towards it in Experiment 2. These results suggest that the Japanese macaques do not respond to (human) gaze in a fixed way, but rather in a context-specific way. This might reflect a sophisticated interpretation of others’ gaze cues that allows macaques to flexibly adapt to the changing conditions of a complex social world.

In the cooperative context (Experiment 2) we did not observe touching gestures, and we did not find differences in the rate of vocalizations between conditions. These findings suggest that, in a feeding context, Japanese macaques, like great apes^38^, prefer moving into others’ visual fields rather than using vocal or touching attention-getter cues (which might instead be used when movements are restricted^37^). A reason for this preference could be that vocalizations can attract the attention of conspecifics^70^, producing possible competitive situations – something especially risky for Japanese macaques, since they are a highly despotic species^71^. Furthermore, physical contact with E1 would be riskier for the macaques than using visual gestures.

These results show that our study subjects paid attention to the human’s head and probably body orientation. Furthermore, they could distinguish between eyes-open and eyes-closed in humans, as they showed more threats and self directed-behaviors (SDB) only when E1 was directly looking at them in Experiment 2. This ability to distinguish between eyes-open and eyes-closed was not found in cooperative studies in which macaques were trained to point to food in a referential way to obtain it^39,40^. Possibly, these differences with previous studies are because our experimental approach required no previous training and used behaviors belonging to the subjects’ natural repertoire. It is worth noting, however, that despite being in a cooperative context where the experimenter provided them with food, subjects in Experiment 2 performed SDB (which are related to anxiety^63^) and even threatened E1, especially in the Eyes open condition. As it happened in the context of threat (Experiment 1), subjects in Experiment 2 may have perceived E1’s direct eye gaze as a threatening cue and not just as a mere attentional cue, as it occurs between conspecifics in this and other primate species^72^. Future studies with macaques should better consider the threatening meaning that direct gaze has for this taxon, and for example substitute the Eyes open condition with a condition in which the experimenter looks forward but not directly to the subjects.

Although in the two first experiments the Japanese macaques showed to be sensitive to the human’s gaze, this does not necessarily mean that this species can represent basic psychological states of others (e.g., intentions and perceptions).  These results may have simpler alternative explanations based on the subjects “reading” the human’s behavior, rather than understanding his mental states^57,58^. For example, subjects in Experiment 1 might have associated direct gaze with danger, or they could have learned that the direction of the others’ gaze usually corresponds to the direction of their movement. In the same way, subjects in Experiment 2 could have simply learned that they are more likely to receive food when frontally seeing E1’s eyes or face. Experiment 3, however, was devised so that E1′s gaze cues could not guide the subjects’ responses. Instead, their behavior could only be affected by the inference that E1 could see the food placed behind the transparent barrier, but not behind the opaque one (after ruling out a baseline preference for one type of barrier in the Control condition, when E1 was not present). Subjects did not show a preference for the opaque barrier (neither in the Experimental nor in the Control condition) in this experiment, despite the task was carried out in a context of competition over food which should elicit a high motivation to behave strategically in primates^48^. Furthermore, although increasing the distance between food rewards should have enhanced subjects’ motivation to choose the most strategic option^52^, we found no difference in subjects’ choices of the opaque barrier when the barriers were closer or further apart.

The results of Experiment 3 can have several explanations. On the one hand, they may suggest that, contrary to our predictions, the macaques in our study were not able to take the visual perspective of a human competitor into account. Although some studies have claimed that other primate species^52^, including macaques^53,54^, can use the visual perspective of conspecifics in competitive situations, their findings could be explained by the subjects responding to the previous behavioral cues performed by the competitors towards the food^57,58^. Thus, it is possible that Japanese macaques are not capable of taking the visual perspective of others into account, or that they cannot do it in the absence of specific attentional cues like head or body orientation towards objects. Alternatively, it may be that taking the visual perspective of a conspecific is easier than taking the visual perspective of a non-conspecific^73^. Another possible explanation is that not all the macaques followed the same strategy in Experiment 3, which could have diluted the results. For example, the faster individuals^54^ or those that were less shy towards humans^74^ may have grabbed first the food “at risk”, to maximize food intake, whereas the slowest or shiest ones may have preferred to first go for the “safer” food, to ensure at least some food intake. Furthermore, it is possible that subjects perceived the transparent barrier as also preventing the human to interfere. Finally, it might be that our experimental procedure failed to mimic a competitive context, thus reducing subjects’ motivation to be strategic in their choices. Even though we increased the distance between the barriers from the first to the second session, it was still relatively easy for the subjects to monopolize the food from both barriers (i.e., they retrieved both food pieces in 97% of the trials.), mainly because E1 never tried to obtain the food in the Experimental trials, to avoid providing monkeys with behavioral cues. Moreover, macaque previous experience with humans in Koshima, where humans may move around the island and provide them with food, but never compete with monkeys over it, might have contributed to this effect, despite the Familiarization trials.

It should be noted that, although our study subjects live freely in their habitat, they are extensively habituated to humans due to the presence of researchers, tourists, and technicians from the Koshima Field Station. Therefore, it is possible that other groups with no contact with humans would have performed differently. In several primate species, exposure to humans can lead to an improvement in the interpretation of human cues^75,76^ as well as a decrease in the defensive response^16^. The latter could explain why our study subjects did not show any difference in the rate of alarm calls and SDB in the context of threat (Experiment 1), and may have also affected subjects’ ability to see the experimenter as a competitor in Experiment 3. In the future, it would be interesting to compare our results with those of other studies carried out with Japanese macaques being less exposed to humans.

In conclusion, this study has shown that Japanese macaques are sensitive to the human gaze. In particular, the macaques in our study used gaze cues to avoid a human in a threat context, and to increase the probability of receiving food in a cooperative feeding context. However, they did not seem to take the human’s visual perspective into account in a competitive context. Importantly, these results were obtained by measuring spontaneous behavior in non-trained wild subjects, tested across different experimental contexts and with a limited number of trials (to avoid learning effects during the experiments). Finally, we could control for the effect of macaques’ age, sex, and rank on their response in these tasks. Hopefully, future research with larger samples will be able to assess whether and how these individual characteristics modulate macaque sensitivity to others’ gaze and visual perspective. Future research should also design more experimental protocols^77^ to investigate whether specific contexts (e.g., threat, cooperation, competition) foster the emergence of mental state attribution in primates, and might have thus been important selective pressures during the evolutionary history of this trait.

## Supplementary Information

Supplementary Information.
