# Supplementary material for: Japanese Macaques’ (Macaca fuscata) sensitivity to human gaze and visual perspective in contexts of threat, cooperation, and competition
Source: Sci Rep. 2021 Mar 4;11:5264. doi: 10.1038/s41598-021-84250-5 (PMC7933183; doi:10.1038/s41598-021-84250-5)
Supplement: Supplementary file 1 — Supplementary Information. [file 41598_2021_84250_MOESM1_ESM.docx]

**Supplementary Information**

# JAPANESE MACAQUES’ (*Macaca fuscata*) SENSITIVITY TO HUMAN GAZE AND VISUAL PERSPECTIVE IN CONTEXTS ­­­­­­­­­­­­­­­­­­­­­­­­­OF THREAT, COOPERATION, AND COMPETITION

Alba Castellano-Navarro, Emilio Macanás-Martínez, Zhihong Xu, Federico Guillén-Salazar, Andrew JJ MacIntosh, Federica Amici, Anna Albiach-Serrano.

**A. Subject information**

***Table A.1.*** Individual characteristics of the subjects who participated in each experiment. F = female, M= male, Exp. = experiment.

| **Subject** | **Age (years)** | **Sex** | **Rank** | **Exp. 1** | **Exp. 2** | **Exp. 3** |
| --- | --- | --- | --- | --- | --- | --- |
| **Takana** | 0 | F | .505 | x |  |  |
| **Botan** | 1 | F | .094 | x |  |  |
| **Mochi** | 1 | F | .165 | x |  |  |
| **Pan** | 1 | F | .153 | x |  |  |
| **Pichi** | 1 | F | .250 | x |  |  |
| **Yotsuba** | 1 | F | .443 | x |  |  |
| **Haku** | 1 | M | .383 | x | x |  |
| **Komatsu** | 1 | M | .315 | x |  |  |
| **Mekki** | 1 | M | .138 | x |  |  |
| **Noko** | 1 | M | .344 | x |  |  |
| **Shida** | 1 | M | .354 | x |  |  |
| **Yumin** | 1 | M | .171 | x |  |  |
| **Betei** | 2 | F | .049 | x |  |  |
| **Hiba** | 2 | F | .197 | x |  |  |
| **Tabu** | 2 | F | .505 | x | x | x |
| **Binega** | 3 | F | 0 | x |  |  |
| **Hado** | 3 | F | .279 | x |  |  |
| **Tsutsuji** | 3 | F | .433 | x |  |  |
| **Bon** | 3 | M | .136 | x |  |  |
| **Kote** | 3 | M | .484 | x | x |  |
| **Shiira** | 3 | M | .388 | x | x | x |
| **Yomogi** | 3 | M | .366 | x |  |  |
| **Yuu** | 4 | M | .283 | x | x | x |
| **Tsuwa** | 5 | F | .485 | x | x | x |
| **Yone** | 6 | F | .413 | x |  | x |
| **Yuna** | 6 | F | .188 | x | x | x |
| **Beni** | 7 | F | .128 | x | x | x |
| **Mizu** | 7 | F | .281 | x | x | x |
| **Buta** | 8 | M | - |  | x | x |
| **Neji** | 8 | M | .404 |  | x | x |
| **Shika** | 8 | M | .647 | x | x | x |
| **Mikan** | 9 | F | .338 | x | x | x |
| **Okura** | 9 | F | .567 | x | x | x |
| **Kizu** | 10 | F | .429 | x | x | x |
| **Keta** | 10 | M | .763 | x | x | x |
| **Okapi** | 10 | M | .531 | x | x | x |
| **Serori** | 11 | F | .250 | x | x | x |
| **Shide** | 11 | F | .705 | x | x | x |
| **Minku** | 11 | M | .537 | x |  |  |
| **Kan'na** | 12 | F | .237 | x | x | x |
| **Kibana** | 12 | F | .501 | x | x | x |
| **Muku** | 12 | F | .219 | x | x | x |
| **Kei** | 12 | M | 1 | x | x | x |
| **Uso** | 12 | M | .504 |  | x | x |
| **Omoto** | 13 | F | .557 | x | x | x |
| **Shiso** | 13 | F | .680 | x | x | x |
| **Kinoko** | 15 | F | .424 | x | x | x |
| **Toga** | 16 | F | .150 | x | x | x |
| **Usu** | 16 | F | .253 | x | x | x |
| **Mushi** | 16 | M | .507 | x | x | x |
| **Yamu** | 18 | F | .656 | x | x | x |
| **Yashi** | 20 | F | .661 | x | x | x |

**B. Ethograms**

***Table B.1.*** Ethogram of agonistic interactions used in calculating the dominance rank. S = subject, P = partner.

| **Dominance behaviors** | |
| --- | --- |
| **Displacement** | S approaches to less than 2 meters from P who, in less than 2 seconds, withdraws. |
| **Threat** | S stares at P with raised eyebrows. This can be accompanied by keeping the mouth half open, growling, tail extended, and/or simulation of attack (*i.e.*, gesture of hitting or starting to chase the other, but without having physical contact or running after the other)^1^. |
| **Persecution** | S runs behind P who runs away, in an agonistic context. |
| **Aggression** | S hits, grabs, pushes, or bites some part of P’s body, in an agonistic context. |
| **Submission behaviors** | |
| **Excretion** | S defecates or urinates, in an agonistic context. |
| **Alarm call/scream** | S makes high and intense sounds, in an agonistic context. This is often accompanied by displaying bared teeth or withdrawing^2,3^. |
| **Bared teeth** | Grimace where S, with the mouth closed, draws back the lips and shows the teeth^4^. The subject can look at P while doing this or turn facing the other way. |
| **Hindquarters presentation** | S orients the anogenital region toward P, generally looking at the other^4^. |

***Table B.2.*** Ethogram used in Experiment 1. S = subject, E1 = experimenter 1.

| **Threat to E1** | S stares at E1 with raised eyebrows. This can be accompanied by keeping the mouth half open, growling, tail extended, and/or simulation of attack^1^. |
| --- | --- |
| **Alarm call** | S makes high and intense sounds^2,3^. |
| **Self-directed behavior (SDB)** | S self-scratches, self-grooms, or self-licks some part of S’s body^5^. |

***Table B.3.*** Ethogram used in Experiment 2. S = subject, E1 = experimenter 1.

| **Moving to the left** | S moves at least the distance of one body to the left. If S waits at least one second and then moves again in the same direction, they are considered two separate events. |
| --- | --- |
| **Moving behind E1** | S moves from zone A (in front of the experimenter) to zone B (behind E1). If S returns to zone A and enters zone B again, they are considered two separate events. |
| **Vocalization** | S makes an ascending melodic sound (from low to high) that progressively increases in intensity during its execution^2,3^. If a second goes by between two sounds, they are considered two separate events. |
| **Threat to E1** | S stares at E1 with raised eyebrows. This can be accompanied by keeping the mouth half open, growling, tail extended, and/or simulation of attack^1^. If S stops for at least one second and repeats the threat, they are considered two separate events. |
| **Self-directed behavior (SDB)** | S rubs the fingers on the hand or foot repeatedly over the fur^5^. If S stops for more than one second and repeats the behavior, they are considered two separate events. |
| **Touching gesture** | S touches E1 with some part of S’s body. |

**REFERENCES**

1. Van Hooff, J. A. R. A. M. The Facial Displays of the Catarrhine Monkeys and Apes. in *Primate ethology* 7–68 (AldineTransaction, 1967).

2. Green, S. Variation of vocal pattern with social situation in the Japanese monkey (*Macaca fuscata*): A field study. in *Primate behavior* (Academic Press, 1975).

3. Itani, J. Vocal communication of the wild Japanese monkey. *Primates* **4**, 11–66 (1963).

4. Maestripieri, D. Gestural communication in three species of macaques (*Macaca mulatta, M. nemestrina, M. arctoides*): Use of signals in relation to dominance and social context. *Gesture* **5**, 57–73 (2005).

5. Maestripieri, D., Schino, G., Aureli, F. & Troisi, A. A modest proposal: displacement activities as an indicator of emotions in primates. *Anim. Behav.* **44**, 967–979 (1992).
